# Rhabdomyolysis Masquerading as Cauda Equina Syndrome

**DOI:** 10.7759/cureus.23048

**Published:** 2022-03-10

**Authors:** Beatrice Tan, Charles Kon, Sean Loh, Louis Ng, Vincent Lum

**Affiliations:** 1 Orthopaedic Surgery, Changi General Hospital, Singapore, SGP; 2 Respiratory and Critical Care Medicine, Changi General Hospital, Singapore, SGP; 3 Anaesthesia and Surgical Intensive Care, Changi General Hospital, Singapore, SGP; 4 Accident and Emergency, Changi General Medicine, Singapore, SGP

**Keywords:** acute urinary retention, acute kidney injury, lower back pain, cauda equina syndrome, rhabdomyolysis

## Abstract

Lower back pain is a very common presenting condition, with a large proportion resulting from discogenic causes, especially after strenuous activity. In patients with a history of exertion, lower back pain, and acute urinary retention, the obvious diagnosis to exclude would be cauda equina syndrome. We present a case of a 32-year-old man who presented with lower back pain, bilateral lower limb weakness, and acute urinary retention following a recent episode of heavy lifting. He was subsequently diagnosed with rhabdomyolysis. This case highlights that rarer conditions can masquerade as cauda equina syndrome, and even in seemingly straightforward presentations, alternative diagnoses should also be considered.

## Introduction

Lower back pain is an extremely common condition and contributes to 3.15% of all presentations to the emergency department in the United States [1]. Discogenic causes account for 39% of cases of lower back pain, with a higher incidence in patients who have physical jobs involving lifting or vibration exposure [2]. However, even in patients with seemingly straightforward presentations, alternative diagnoses still need to be considered. We present a patient with lower back pain associated with bilateral lower limb weakness secondary to rhabdomyolysis of bilateral gluteal and lower back muscles.

## Case presentation

A 32-year-old man presented to the emergency department (ED) with a one day history of lower back pain after heavy lifting of 30 kg at work. He had associated bilateral lower limb weakness and numbness, although he did not report any radicular pain. On initial presentation, he claimed not to have any urinary or bowel symptoms. He did not have any significant past medical history apart from previous intravenous drug use, although he denies any recent use. Investigations on admission revealed that he had a stage 3 acute kidney injury (AKI) with creatinine of 403 µmol/L, severe hyponatraemia of 127 mmol/L, and hyperkalaemia of 6.2 mmol/L (Table 1).

**Table 1:** Blood test results on admission

	Lab results	Reference ranges
Sodium	127 mmol/L	135-145 mmol/L
Potassium	6.2 mmol/L	3.5-5.3 mmol/L
Creatinine	403 µmol/L	65-125 µmol/L
Urea	9.6 mmol/L	2.8-7.7 mmol/L
Bicarbonate	21 mmol/L	19-31 mmol/L
Creatine kinase	67,186 U/L	40-210 U/L

**Figure 1:**
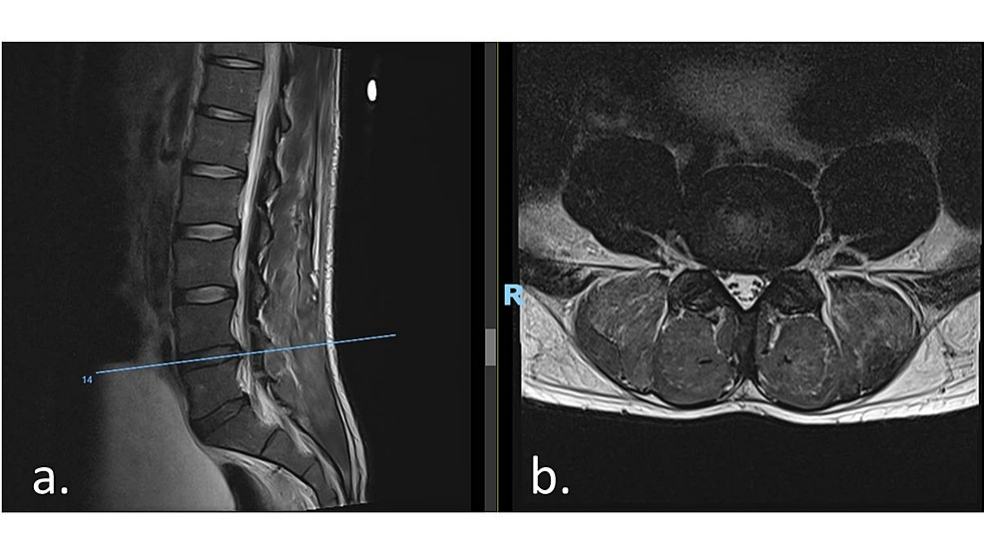
MRI lumbar spine showing an L4/5 diffuse disc bulge, with mild bilateral subarticular zone stenosis with possible mild compression of the left descending L5 nerve root A distended urinary bladder is noted. (a) Sagittal cut. (b) Axial cut. MRI: magnetic resonance imaging, R: right.

**Figure 2:**
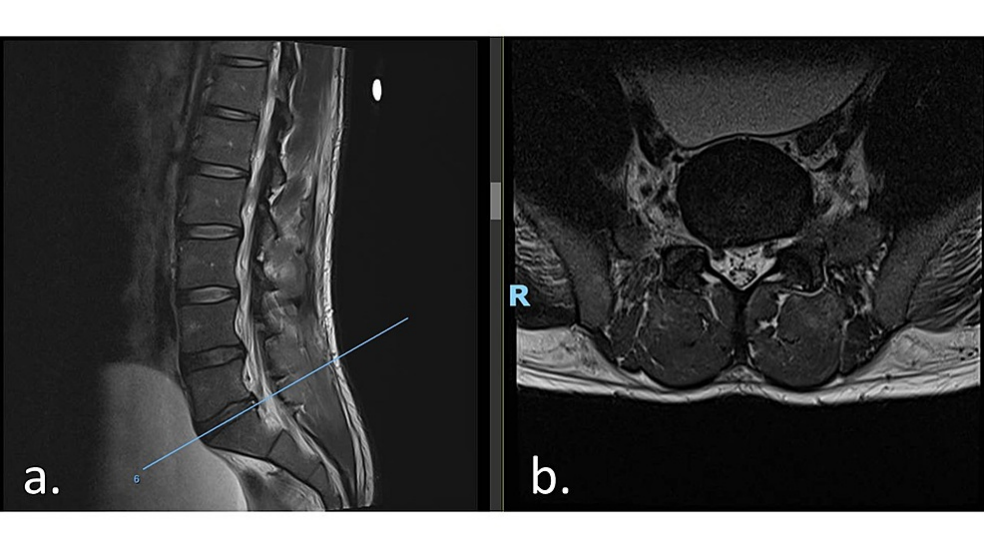
MRI lumbar spine showing L5/S1 diffuse disc bulge with a right paracentral annular fissure, mild right subarticular zone stenosis, with the disc contacting the right descending L5 nerve root (a) Sagittal cut. (b) Axial cut. MRI: magnetic resonance imaging, R: right.

A bedside ultrasound performed in ED also showed a distended bladder. An urgent magnetic resonance imaging (MRI) of his lumbar spine was carried out on the same day for a working diagnosis of cauda equina syndrome secondary to a prolapsed intervertebral disc. The MRI showed a diffuse L4/5 disc bulge with mild left descending L5 nerve root compression (Figures 1a, 1b) and the L5/S1 disc contacting the right descending L5 nerve root. The distended urinary bladder was again noted, although there was no compression of the cauda equina (Figures 2a, 2b).

**Figure 3:**
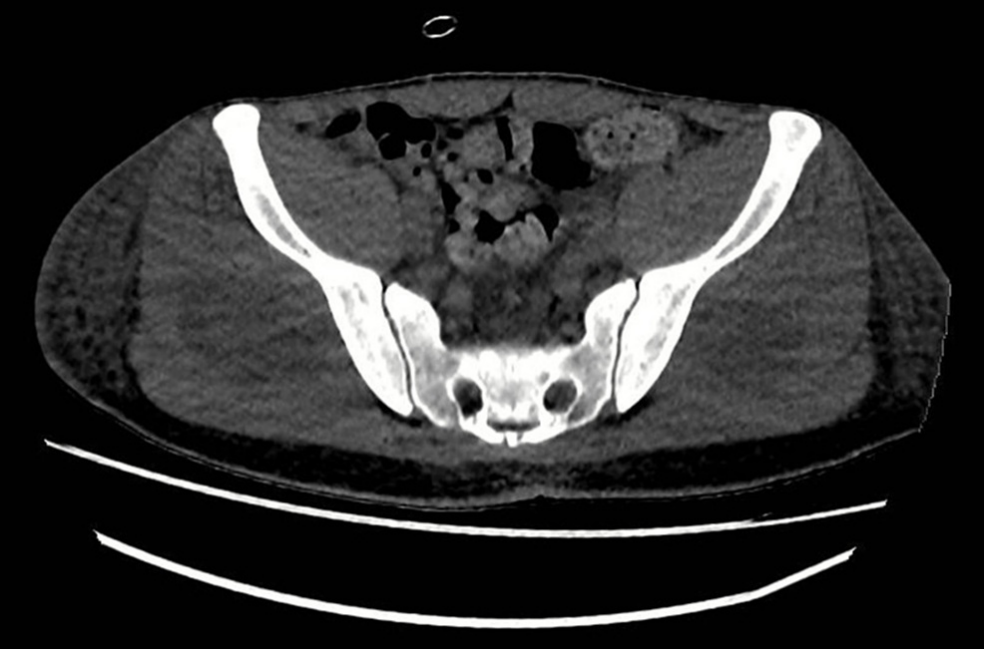
CT KUB showing swelling of the bilateral gluteus medius and minimus muscles with faint hypodense areas within, with overlying subcutaneous fat stranding and oedema CT KUB: computed tomography of the kidneys, ureters, and bladder.

His creatine kinase (CK) was found to be significantly raised at 67,186 U/L (normal range: 40-210 U/L) (Table 1). Computed tomography of the kidneys, ureters, and bladder (CT KUB) confirmed rhabdomyolysis with acute myositis involving bilateral gluteal and lower back muscles with surrounding inflammatory change. Bilateral kidneys were normal, and there were no features of obstructive uropathy (Figure 3).

His renal function unfortunately continued to deteriorate over the next few days despite starting a fluid regime for rhabdomyolysis, and he eventually developed anuria requiring admission to the surgical intensive care unit (SICU) for renal replacement therapy (RRT). He underwent two weeks of RRT, following which his creatinine levels stabilised and urine output improved. He was eventually discharged after three weeks of inpatient management. He returned to normal walking, and no longer required dialysis on discharge.

## Discussion

This patient presented to the emergency department with what appeared to be a typical presentation of discogenic lower back pain with likely cauda equina syndrome. He had developed lower back pain following recent heavy lifting, which was associated with bilateral lower limb weakness and numbness, and was also found to be in acute urinary retention on admission. However, workup for the initial presumptive diagnosis of cauda equina syndrome had turned out to be negative. The final diagnosis of rhabdomyolysis was eventually made on the basis of his markedly raised CK, which was identified on further investigation for his AKI and deranged electrolytes. This diagnosis was also subsequently confirmed on CT KUB.

There have been multiple case reports in the literature of rhabdomyolysis as an unusual case of lower back pain. Wik et al. [3] reported on five cases of young males aged 24 to 37 years old with active lifestyles, and all similarly had a history of strenuous physical activity immediately prior to presentation with severe lumbosacral pain. These cases were also noted to have raised CK, liver function tests, lactate dehydrogenase, and white cell counts. Three of these patients required surgical fasciotomies, but made full recovery, whereas the remaining two were managed conservatively with residual mild chronic back pain with vigorous exercise. In comparison of previously reported cases as consolidated by Minnema et al., there have also been multiple reports of sensory loss over the affected muscles [4].

Compartment syndrome as a known complication of paraspinal rhabdomyolysis had also been reported in multiple cases [5]. The CT KUB report in our patient highlighted acute myositis and surrounding inflammatory changes, however, did not specifically report on the presence of muscle swelling. There was also no measurement of compartment pressure in our patient.

This patient was also noted to have an AKI on presentation. This is a serious complication of rhabdomyolysis. Particularly in those with additional risk factors such as this patient, acute renal failure may develop, necessitating dialysis as in his case. Exercise-associated acute renal failure complicates 4%-33% of cases of patients with rhabdomyolysis [6]. There is a relatively higher morbidity and mortality rate in patients who develop AKI as a result of rhabdomyolysis, with a reported mortality rate ranging from 7% to 20%. In patients requiring dialysis, this rate increases up to 50% [6]. This emphasizes the importance of considering atypical causes of AKI such as rhabdomyolysis, particularly in young patients with a background of musculoskeletal insult, to allow for early management with aggressive hydration.

Overexertion or prolonged strenuous activity is a well-recognised physical cause of rhabdomyolysis and, with the increase in exercise trends of spinning and high interval intensity training (HIIT), is likely to present more frequently. However, it can also result from multiple other causes such as burns or trauma, chemical causes like alcohol or drug use, both illicit and prescribed, or biological causes like infections, inflammatory conditions, or endocrine diseases [6]. This patient had a few risk factors for developing rhabdomyolysis. Besides his history of recent heavy lifting, he also has a history of intravenous heroin use. However, he claims that he has abstained for the last two years, and both his urine and serum drug screens were negative. He was also noted to have hyponatraemia and hyperkalaemia on admission. Hyperkalaemia has also been associated with rhabdomyolysis [7]. Considering these various factors, development of rhabdomyolysis in our patient is more likely due to recent overexertion, although possible drug exposure may have increased his susceptibility to developing rhabdomyolysis. Our patient did not require fasciotomy or undergo any surgical intervention for his condition.

## Conclusions

Not all patients presenting with a typical history of recent exertion have discogenic causes of back pain. In particular, in patients with a triad of exertional history, lower back pain, and acute urinary retention, the initial emergency to exclude would undoubtedly be acute cauda equina syndrome. However, this case demonstrates that rhabdomyolysis can easily masquerade as cauda equina syndrome. It is worth considering the possibility of rarer differentials, especially in patients presenting with an AKI, deranged electrolytes, and markedly raised CK levels.
